# A study on demographic and psychiatric suicide risk factors and their correlation in the community dwelling elderly

**DOI:** 10.1192/j.eurpsy.2023.779

**Published:** 2023-07-19

**Authors:** K. Lee

**Affiliations:** Psychiatry, Dongguk University Hospital, Gyeongju, Korea, Republic Of

## Abstract

**Introduction:**

According to data from the National Statistical Office of the Republic of Korea, the number of suicides increased with increasing age, and the elderly over the age of 65 had a higher suicide success rate using lethal means.

Among mental disorders, depression is known to be the most associated with suicide, and suicidal thoughts help predict the risk of suicide. Dementia, depression, and sleep disorders, which are typical mental health problems of the elderly, require treatment, but only 10% of the elderly receive appropriate treatment at the right time.

**Objectives:**

The purpose of this study was to identify suicide risk factors among the community dwelling elderly and to reveal their correlations. In addition, the differences of suicide risk factors were analyzed in the cognitively impaired group and the cognitively normal group.

**Methods:**

We investigated 20,127 elderly over aged 65, from January 2019 to December 2019. The participants were asked to complete questionnaires. Cognitive function, depression, anxiety, sleep disturbance, suicidal idea data was obtained by mini-mental status examination for dementia screening (MMSE-DS), short geriatric depression scale (SGDS), geriatric anxiety inventory (GAI), Athens insomnia scale (AIS), and scales for suicidal ideation (SSI). We used the Chi-squared test and logistic regression analysis for these data to examine the suicidal risk factors and to analyze the relationships. And differences in suicide risk factors according to cognitive function were also analyzed.

**Results:**

Age, cognitive function, depression, anxiety, and sleep disturbance were identified as risk factors for suicide among the community dwelling elderly. Depression was the factor that increase the risk of suicide the most, followed by anxiety, impaired cognitive function, sleep disturbance, the late elderly (85 years or older), and the middle aged elderly (75-84 years old). In addition, depression increased the risk of suicide by 1.86 times in the cognitively impaired group.

**Image:**

**Figure:**
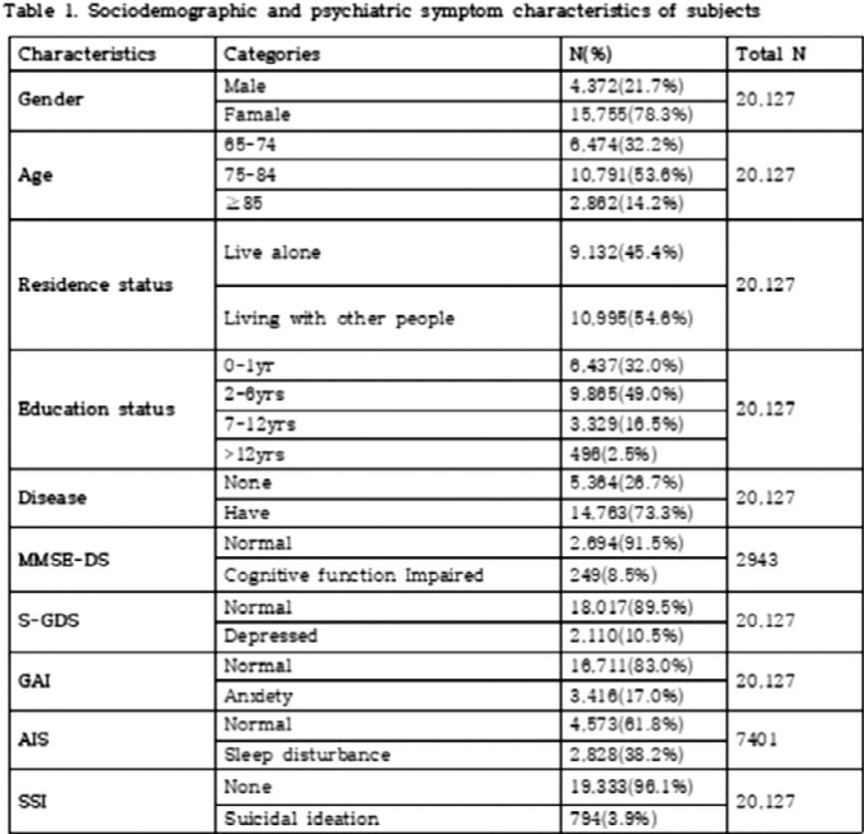

**Image 2:**

**Figure:**
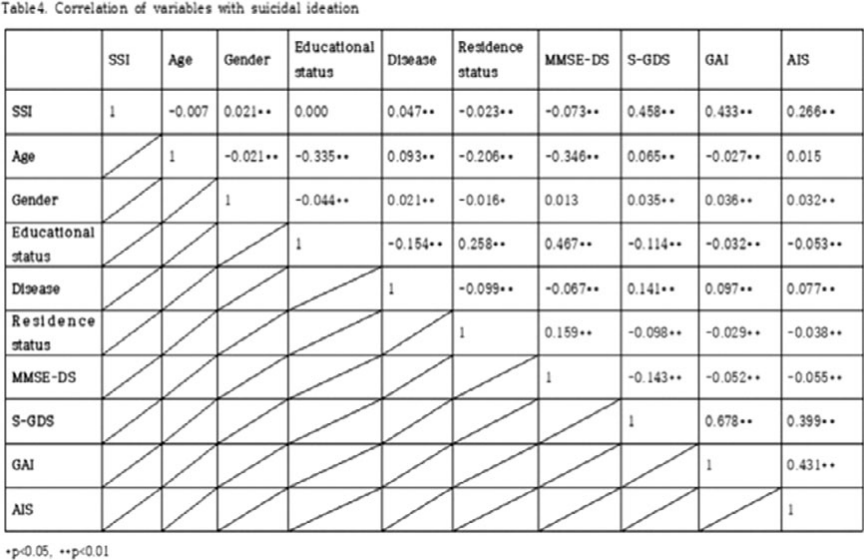

**Image 3:**

**Figure:**
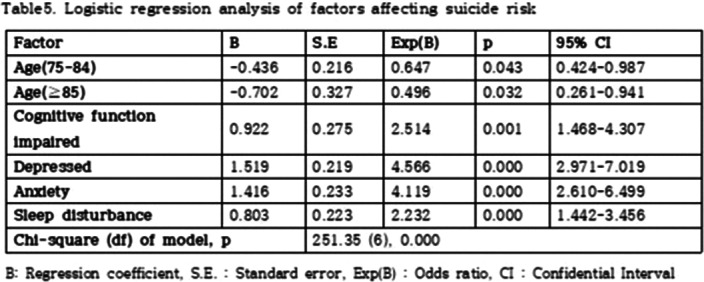

**Conclusions:**

Among community dwelling elderly, depression was the most contributing suicide risk factor. Prevention and treatment of depressive symptoms should be more active in the cognitively impaired group.

**Disclosure of Interest:**

None Declared

